# Supplementary material for: Computational design of soluble and functional membrane protein analogues
Source: Nature. 2024 Jun 19;631(8020):449–58. doi: 10.1038/s41586-024-07601-y (PMC11236705; doi:10.1038/s41586-024-07601-y)
Supplement: Supplementary file 1 — Supplementary methods, references, Figs. 1–10 and Table 1. [file 41586_2024_7601_MOESM1_ESM.pdf]

nature portfolio

https://doi.org/10.1038/s41586-024-07601-y

Supplementary information

Computational design of soluble and functional membrane protein analogues

In the format provided by the authors and unedited

Supplementary Methods

Design solubility and binding screen

All experimentally tested designs are listed in Supplementary Table 2. Designs were synthesized as gene fragments by Twist Bioscience and cloned between the NheI and XhoI sites in pET21b vector with C-terminal His_6-tag. Designs were transformed into *E. coli* BL21 DE3 cells and expressed overnight with 1 mM IPTG at 18 ºC in 1 ml LB medium supplemented with 100 µg/ml ampicillin in a 96-well format. After expression, cells were chemically lysed, centrifuged, and the supernatant was incubated with 50 µl of equilibrated Ni-NTA agarose (QIAGEN) beads. Supernatant was discarded, beads were washed 5 times with 50 mM Tris-HCl pH 7.5, 500 mM KCl, 15 mM imidazole, and designs were eluted in 50 mM Tris-HCl pH 7.5, 500 mM KCl, 400 mM imidazole. Elutions were analyzed using SDS-PAGE and designs were denoted soluble if the appropriate protein band was clearly visible following Coomassie staining. For binding screens, Pierce™ Protein A/G Agarose (Thermo Scientific) beads were used instead, and imidazole is omitted from the washing step. Ten µg of Fab were added to each lysate, allowing to pull-down interacting constructs. Complexes were eluted with 0.1 M glycine pH 2.5 and analyzed using SDS-PAGE.

Protein expression, purification and characterization

Designed proteins were expressed in *E. coli* BL21 (DE3) (Novagen) for 16 h at 18 °C. Bacterial pellets were resuspended and sonicated in 50 mM Tris-HCl pH 7.5, 500 mM KCl, 15 mM imidazole, 1 mg/ml lysozyme, 1 mM PMSF, and 1 µg/ml DNase. Cell lysates were clarified using ultracentrifugation and loaded on a 10 ml Ni-NTA Superflow column (QIAGEN) and washed with 7 column volumes of 50 mM Tris-HCl pH 7.5, 500 mM NaCl, 10 mM imidazole. Designs were eluted with 10 column volumes of 50 mM Tris-HCl pH 7.5, 500 mM NaCl, 500 mM imidazole. Main protein fractions were concentrated and injected onto a Superdex 75 16/600 gel filtration column (GE Healthcare) in PBS. CLN4_20 for cryoEM studies was purified in 20 mM HEPES pH 8.0, 150 mM NaCl using a S200 10/300 GL column (GE Healthcare). Protein fractions were concentrated, flash frozen in liquid nitrogen, and stored at -80 °C. Molar mass and homogeneity were confirmed using SEC-MALS. Folding, secondary structure content, and melting temperatures were assessed using circular dichroism in a Chirascan V100 instrument from Applied Photophysics. miniGs construct 414 was expressed and purified as described previously[59].

Antibody and Fab expression and purification

IgG antibodies and Fabs were expressed in 25 ml cultures of Expi293 cells with Invitrogen ExpiFectamine™ 293 Transfection Kit (A14525) following supplier's recommendations. After 6 days of secretion, the cell culture supernatant was collected, loaded on a 5 ml Ni-NTA Superflow column (QIAGEN), and eluted in 50 mM Tris-HCl pH 7.5, 500 mM NaCl, 500 mM imidazole buffer. The eluate was purified using a Superdex 200 16/600 gel filtration column in PBS. The protein eluted as a single peak at expected retention volume. Collected fractions were concentrated to 1 mg/ml, flash frozen in liquid nitrogen, and stored at -80 °C.

Molecular dynamics-based backbone perturbation

All molecular dynamics (MD) simulations were performed using the Groningen Machine for Chemical Simulations (GROMACS) 2021.4 software package[78]. For the unbiased simulations in water, the Amber ff99SB-ILDN force field for proteins[79] was used. The simulations were carried out under *NPT* conditions with a leapfrog integration scheme and a time step of 2.0 fs. Rhombic dodecahedron (triclinic) periodic boundary conditions were applied and the TIP3P[79] water model was used as solvent. Temperature coupling using stochastic velocity rescaling to two separate temperature baths for the protein and for the water solvent was applied with a reference temperature of 300 K and a relaxation time of 0.1 ps. The pressure was coupled isotropically to a Parrinello-Rahman barostat at 1.0 bar with a coupling constant of 2.0 ps and an isothermal compressibility of 0.45 nm^2 N^{-1}. For both the short-range electrostatic- and van der Waals interactions, a single cutoff distance of 0.9 nm was used. The long-range electrostatics were calculated by the particle mesh Ewald (PME) algorithm with a Fourier spacing of 0.12 nm. The linear constraint solver (LINCS) algorithm was used to impose constraints on the bond lengths with fourth order expansion. Preceding the simulations, the solvated protein structures were energy minimized with a steepest descent algorithm, until the maximum force was below 100 kJ mol^{-1} nm^{-1}. For the unbiased simulations in the POPC lipid bilayer (a mimic for a cellular membrane), the GROMOS 54A8 force field was used in combination with lipid parameters from the 1-Palmitoyl-2-oleoylphosphatidylcholine (POPC) model of Marzuoli *et al.*[80]. The simulations were carried out under *NPT* conditions with a leapfrog integration scheme and a time step of 2 fs. Rectangular periodic boundary conditions were applied and the SPC water model was used as solvent. Nose-Hoover temperature coupling was applied to two separate temperature baths at 323 K, one for the

protein and the POPC bilayer and one for the solvent, with a relaxation time of 0.5 ps. The pressure was coupled semi-isotropically to a Parrinello-Rahman barostat at 1.0 bar with a coupling constant of 2.0 ps and an isothermal compressibility of 0.45 nm^2 N^{-1} was applied. For both the short-range electrostatic and van der Waals interactions, a single cutoff of 1.2 nm was used. The long-range electrostatics were treated using the PME algorithm with a Fourier spacing of 0.16 nm. The bond lengths were constrained with the linear constraint solver (LINCS) algorithm. The simulation systems contained 512 POPC molecules in a bilayer (256 per leaflet). These lipids were packed around the embedded protein structures in the center of the simulation box (2.4963 nm x 13.0172 nm x 13.7189) using the InflateGRO methodology [KANDT2007475], as described by Lemkul[81]. Center-of-mass (COM) motion removal was applied in every simulation step to remove the motion of the bilayer and protein relative to the solvent. Preceding the simulations, the solvated simulation systems were energy minimized with a steepest descent algorithm, until the maximum force was below 1000 kJ mol^{-1} nm^{-1}. For all simulations, the energy minimization was followed by 100 ps *NVT* thermalisation and 1.0 ns *NPT* equilibration. After equilibration, initial velocities were generated using a random number generator. Three unbiased MD runs were performed for 11 ns each and the first 1.0 ns of each simulation was discarded, resulting in 30 ns of sampling for each starting structure. Trajectory frames were extracted every 100 ps.

Supplementary References

61. Suzuki, H. et al. Crystal Structure of a Claudin Provides Insight into the Architecture of Tight Junctions. Science **344**, 304–307 (2014).
62. Christopher, J. A. et al. Structure-Based Optimization Strategies for G Protein-Coupled Receptor (GPCR) Allosteric Modulators: A Case Study from Analyses of New Metabotropic Glutamate Receptor 5 (mGlu5) X-ray Structures. *J Med Chem* **62**, 207–222 (2019).
63. Kingma, D. P. & Ba, J. Adam: A Method for Stochastic Optimization. Arxiv (2014) doi:10.48550/arxiv.1412.6980.
64. Hornak, V. et al. Comparison of multiple Amber force fields and development of improved protein backbone parameters. *Proteins: Struct., Funct., Bioinform.* **65**, 712–725 (2006).
65. Case, D. A. et al. The Amber biomolecular simulation programs. *J. Comput. Chem.* **26**, 1668–1688 (2005).
66. Cock, P. J. A. et al. Biopython: freely available Python tools for computational molecular biology and bioinformatics. *Bioinformatics* **25**, 1422–1423 (2009).
67. Zhang, Y. & Skolnick, J. TM-align: a protein structure alignment algorithm based on the TM-score. Nucleic Acids Res 33, 2302–2309 (2005).
68. Kempen, M. van et al. Fast and accurate protein structure search with Foldseek. *Nat Biotechnol* 1–4 (2023) doi:10.1038/s41587-023-01773-0.
69. Woolfson, D. N. A Brief History of De Novo Protein Design: Minimal, Rational, and Computational. J Mol Biol **433**, 167160 (2021).
70. Sillitoe, I. et al. CATH: increased structural coverage of functional space. Nucleic Acids Res 49, D266–D273 (2020).
71. Vonrhein, C. et al. Data processing and analysis with the autoPROC toolbox. *Acta Crystallogr Sect D Biological Crystallogr* **67**, 293–302 (2011).
72. Liebschner, D. et al. Macromolecular structure determination using X-rays, neutrons and electrons: recent developments in Phenix. *Acta Crystallogr Sect D Struct Biology* **75**, 861–877 (2019).
73. Emsley, P., Lohkamp, B., Scott, W. G. & Cowtan, K. Features and development of Coot. Acta Crystallogr Sect D Biological Crystallogr **66**, 486–501 (2010).
74. Williams, C. J. et al. MolProbity: More and better reference data for improved all-atom structure validation. Protein Sci 27, 293–315 (2018).
75. Pettersen, E. F. et al. UCSF ChimeraX: Structure visualization for researchers, educators, and developers. Protein Sci 30, 70–82 (2021).
76. Orlando, B. J. et al. Development, structure, and mechanism of synthetic antibodies that target claudin and Clostridium perfringens enterotoxin complexes. *J. Biol. Chem.* **298**, 102357 (2022).
77. Kidmose, R. T. et al. Namdinator – automatic molecular dynamics flexible fitting of structural models into cryo-EM and crystallography experimental maps. *IUCrJ* **6**, 526–531 (2019).
78. Lindahl, E., Hess, B. & Spoel, D. van der. GROMACS 3.0: a package for molecular simulation and trajectory analysis. *J Mol Model* **7**, 306–317 (2001).
79. Lindorff-Larsen, K. et al. Improved side-chain torsion potentials for the Amber ff99SB protein force field: Improved Protein Side-Chain Potentials. *Proteins Struct Funct Bioinform* **78**, 1950–1958 (2010).
80. Marzuoli, I., Margreitter, C. & Fraternali, F. Lipid Head Group Parameterization for GROMOS 54A8: A Consistent Approach with Protein Force Field Description. *J Chem Theory Comput* **15**, 5175–5193 (2019).
81. Lemkul, J. From Proteins to Perturbed Hamiltonians: A Suite of Tutorials for the GROMACS-2018 Molecular Simulation Package [Article v1.0]. *Living J Comput Mol Sci* **1**, (2019).

Supplementary Figures

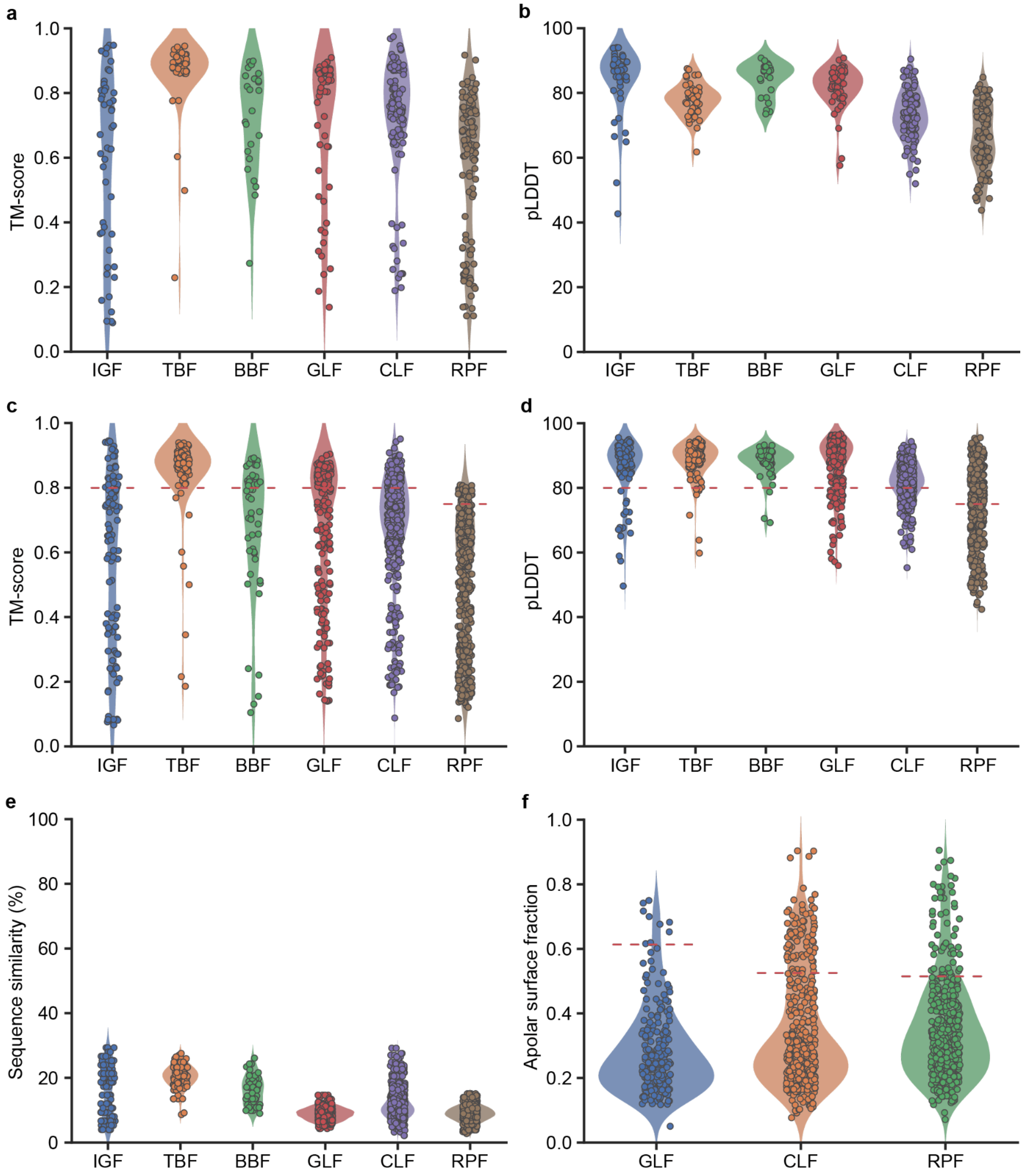

Supplementary Figure 1 | *In silico* analysis of the design folds. **a**, shows the TM-scores of and **b**, confidence of the $AF2_{seq}$ generated sequences. The Ig-like fold (IGF), TIM-barrel fold (TBF) and β-barrel fold (BBF) are designed with ProteinMPNN whilst the GPCR-like fold (GLF), claudin like fold (CLF) and rhomboid protease fold (RPF) are designed with ProteinMPNN version trained on soluble proteins ($MPNN_{sol}$). The models of the $AF2_{seq}$-MPNN sequences are predicted using AF2 and the **c**, TM-scores relative to the designed model and **d**, confidence scores are shown. The dotted line depicts the cutoff values used for in vitro validation filtering. **e**, Plots the sequence similarity between the $AF2_{seq}$-MPNN designed sequence and original design target sequence. **f**, Fraction of apolar surface residues of the $AF2_{seq}$-$MPNN_{sol}$ designs. The dotted line represents the apolar surface fraction of each of the membrane protein design targets.

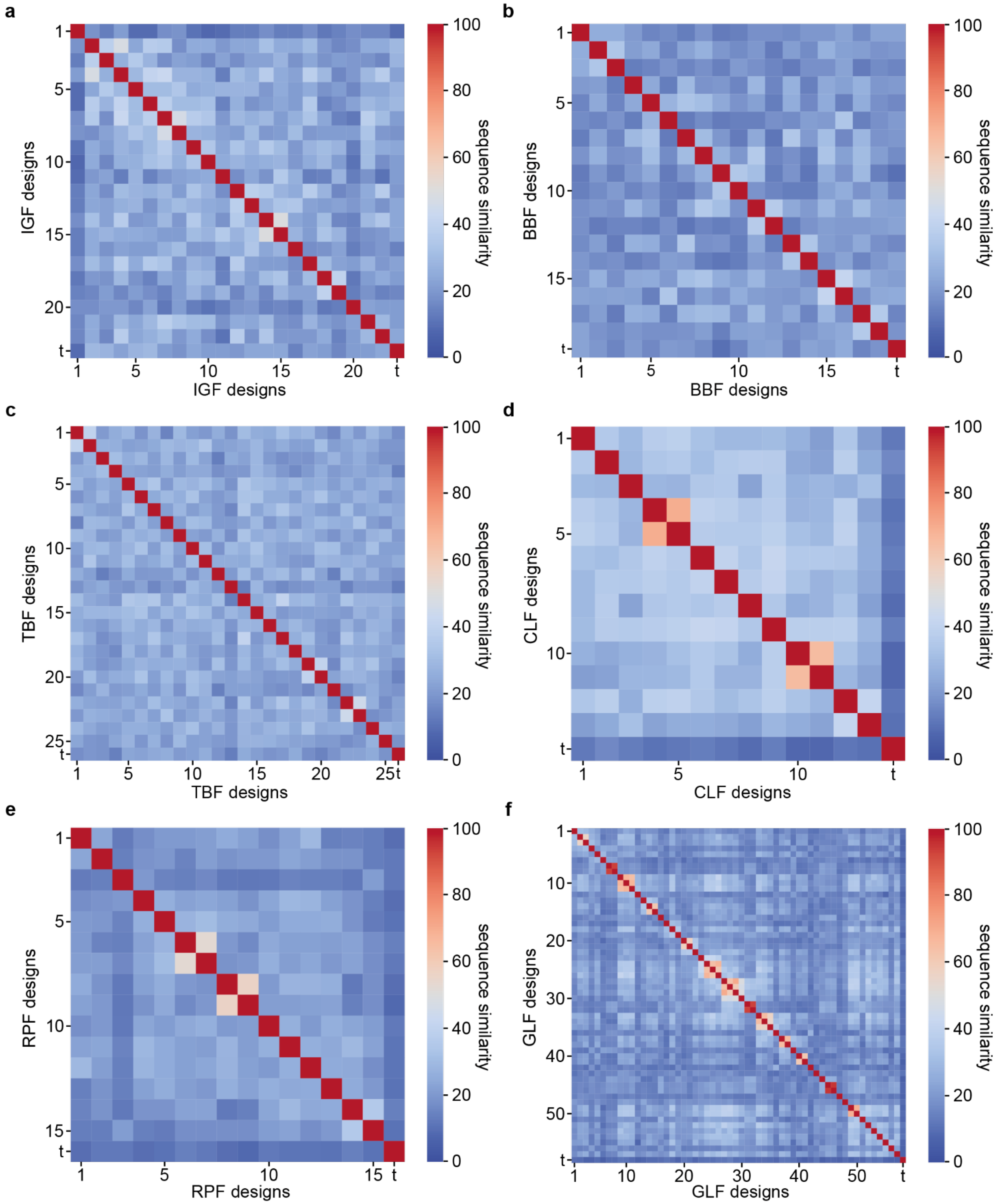

Supplementary Figure 2 | Sequence diversity of the generated designs. Pairwise sequence similarities between **a,** Ig-Like Folds (IGF), **b,** β-barrel Folds (BBF), **c,** TIM-barrel Folds (TBF), **d,** Claudin-Like Folds (CLF), **e,** Rhomboid Protease Folds (RPF), and **f,** GPCR-Like Folds (GLF).

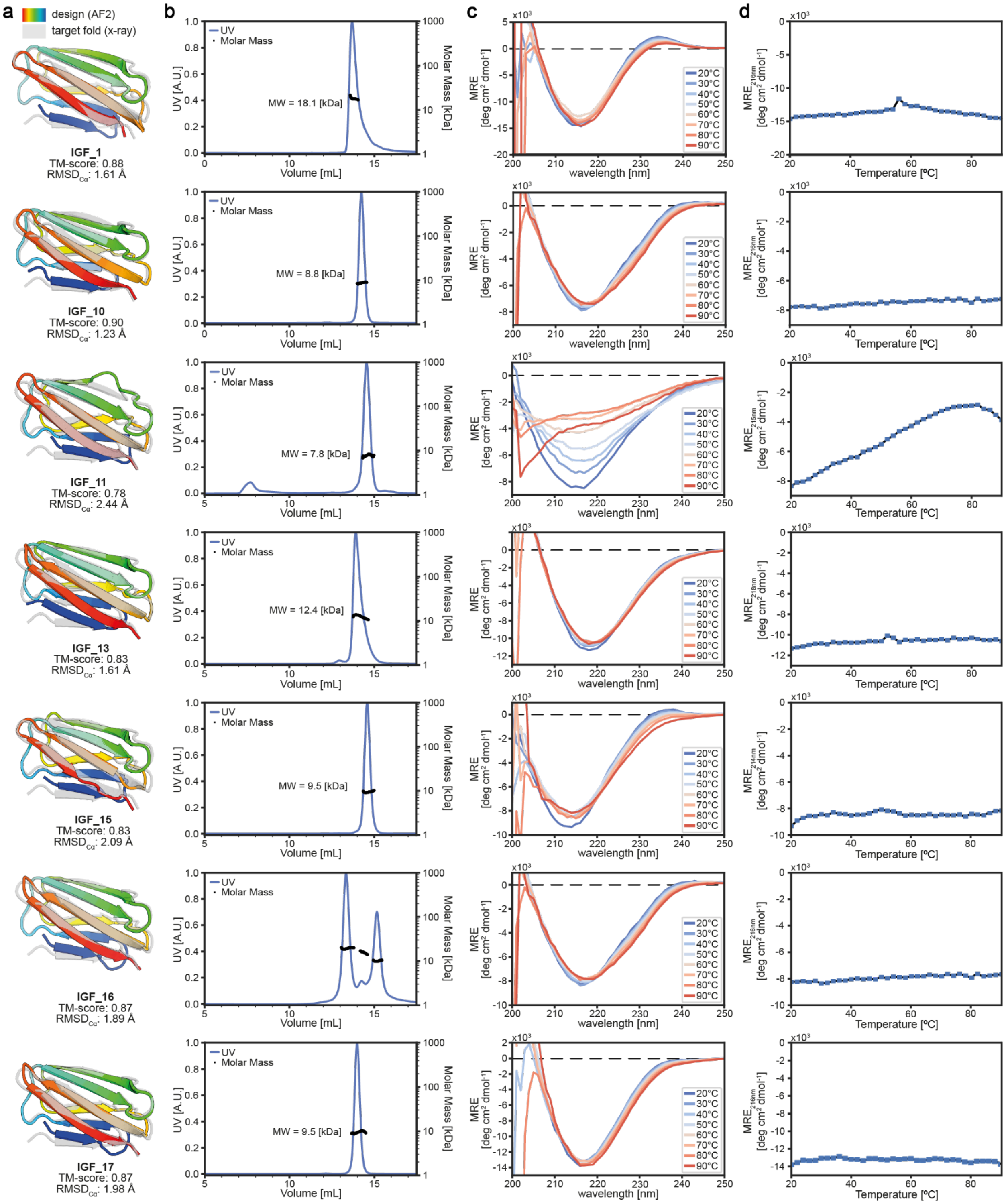

Supplementary Figure 3 | Biophysical characterization of designed Ig-like folds (IGF). a, Cartoon depiction of design (colored) overlaid on the target fold (gray). **b,** SEC-MALS analysis of corresponding design in panel **a**. The expected Mw for the monomeric design ranges from 8.3 to 9.6 kDa. **c,** CD spectroscopy measurements at different temperatures. **d,** Thermostability based on CD measurement.

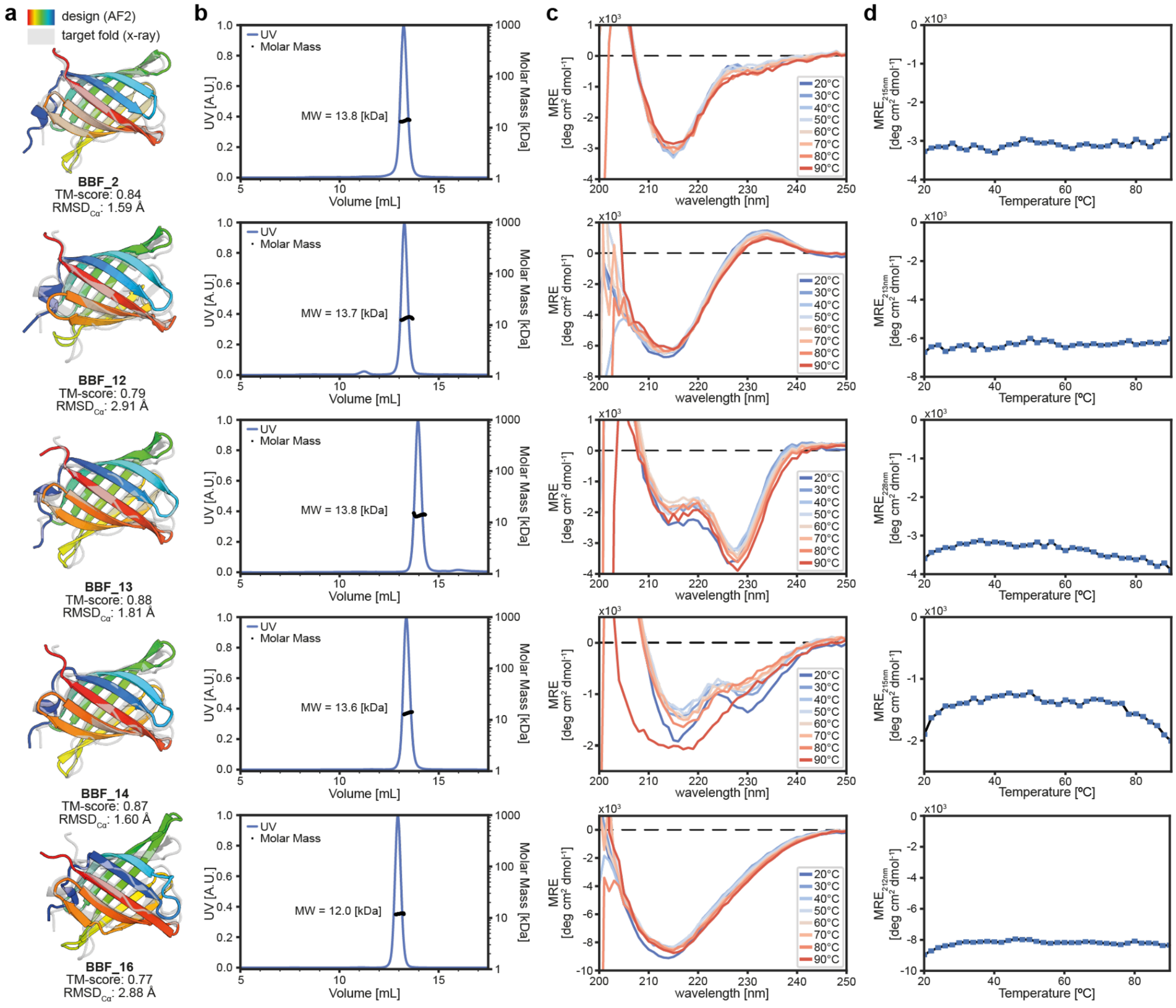

Supplementary Figure 4 | Biophysical characterization of designed β-barrel folds (BBF). a, Cartoon depiction of design (colored) overlaid on the target fold (gray). **b,** SEC-MALS analysis of corresponding design in panel a. The expected Mw for the monomeric design ranges from 12.6 to 13.3 kDa. **c,** CD spectroscopy measurements at different temperatures. BBF_13 and BBF_14 present significant differences in their CD spectra as compared to the expected spectrum of the folded target structure. **d,** Thermostability curve based on CD measurement.

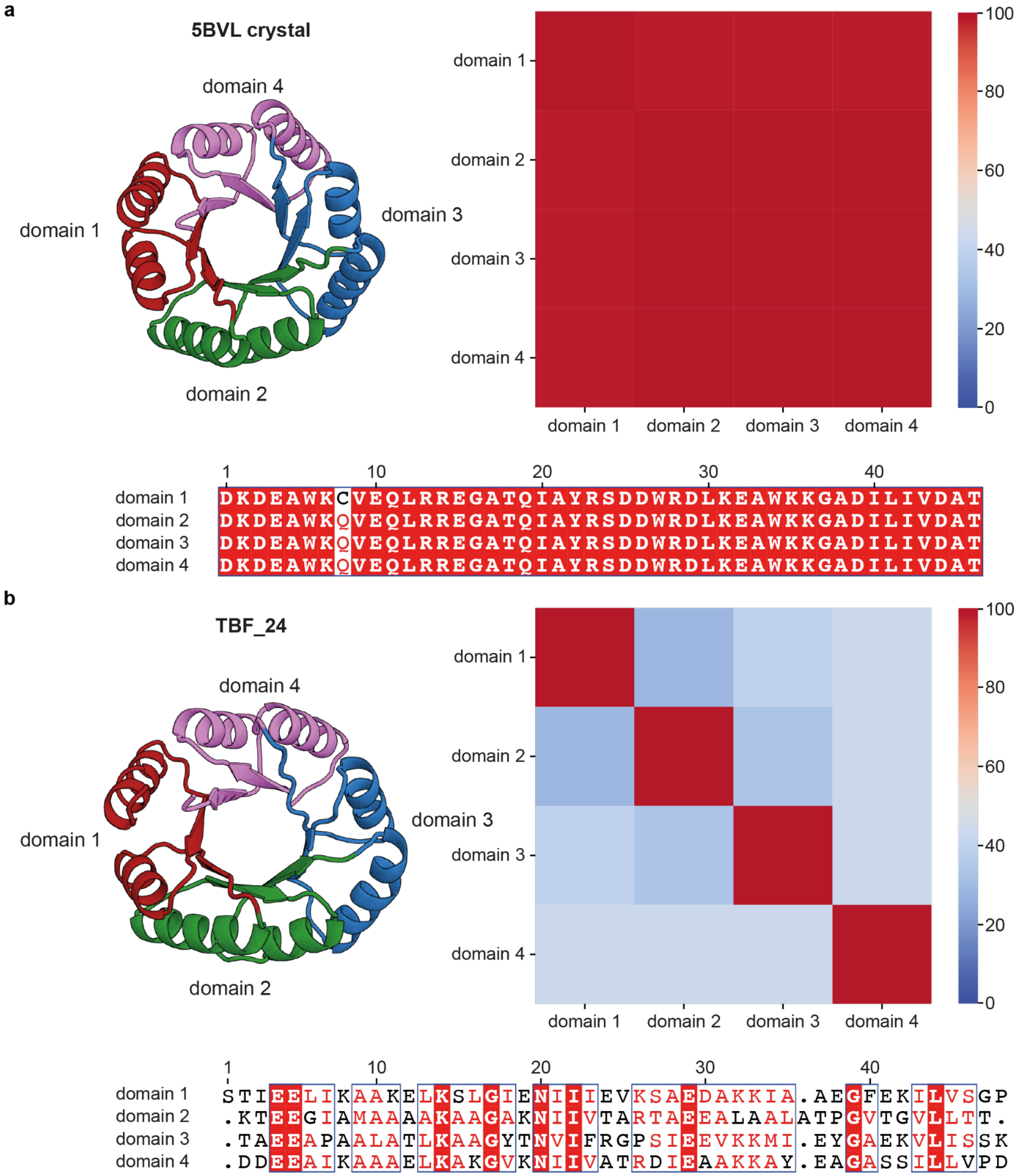

Supplementary Figure 5 | Sequence comparison of *de novo* designed TIM barrels. a, Sequence similarity between the TIM barrel domain segments of the 4-fold symmetric design by Huang et al. **b,** Sequence similarity of the design TBF_24 without symmetric constraints. Crystal structure and sub-domain mapping on the left and sequence identity of the domains on the right.

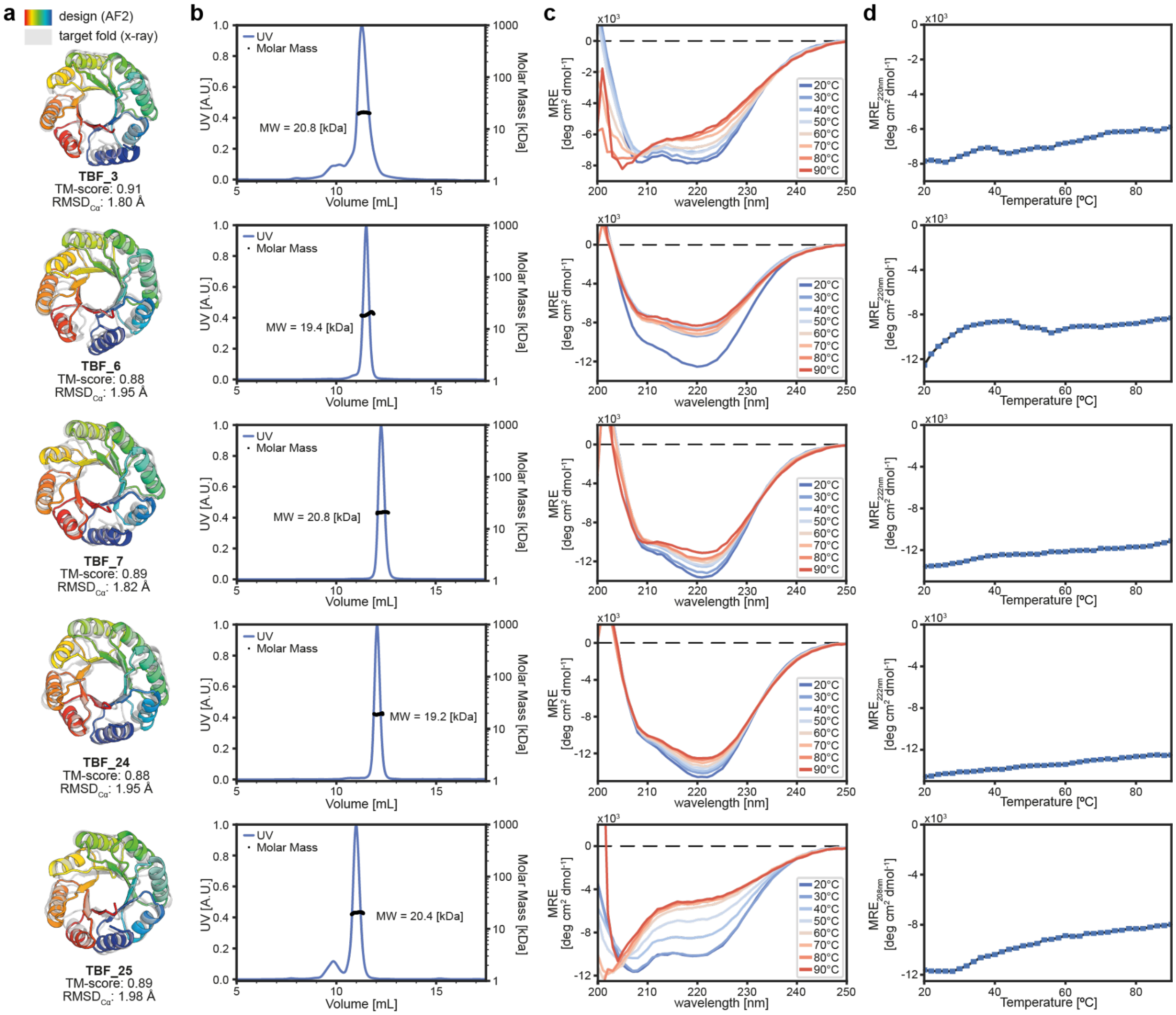

Supplementary Figure 6 | Biophysical characterization of designed TIM-barrel folds (TBF). a, Cartoon depiction of design (colored) overlaid on the target fold (gray). **b,** SEC-MALS analysis of corresponding design in panel a. The expected Mw for the monomeric design ranges from 20.6 to 21.7 kDa. **c,** CD spectroscopy measurements at different temperatures. TBF_3 and TBF_25 present significant differences in their CD spectra as compared to the expected spectrum of the folded target structure. **d,** Thermostability curve based on CD measurement.

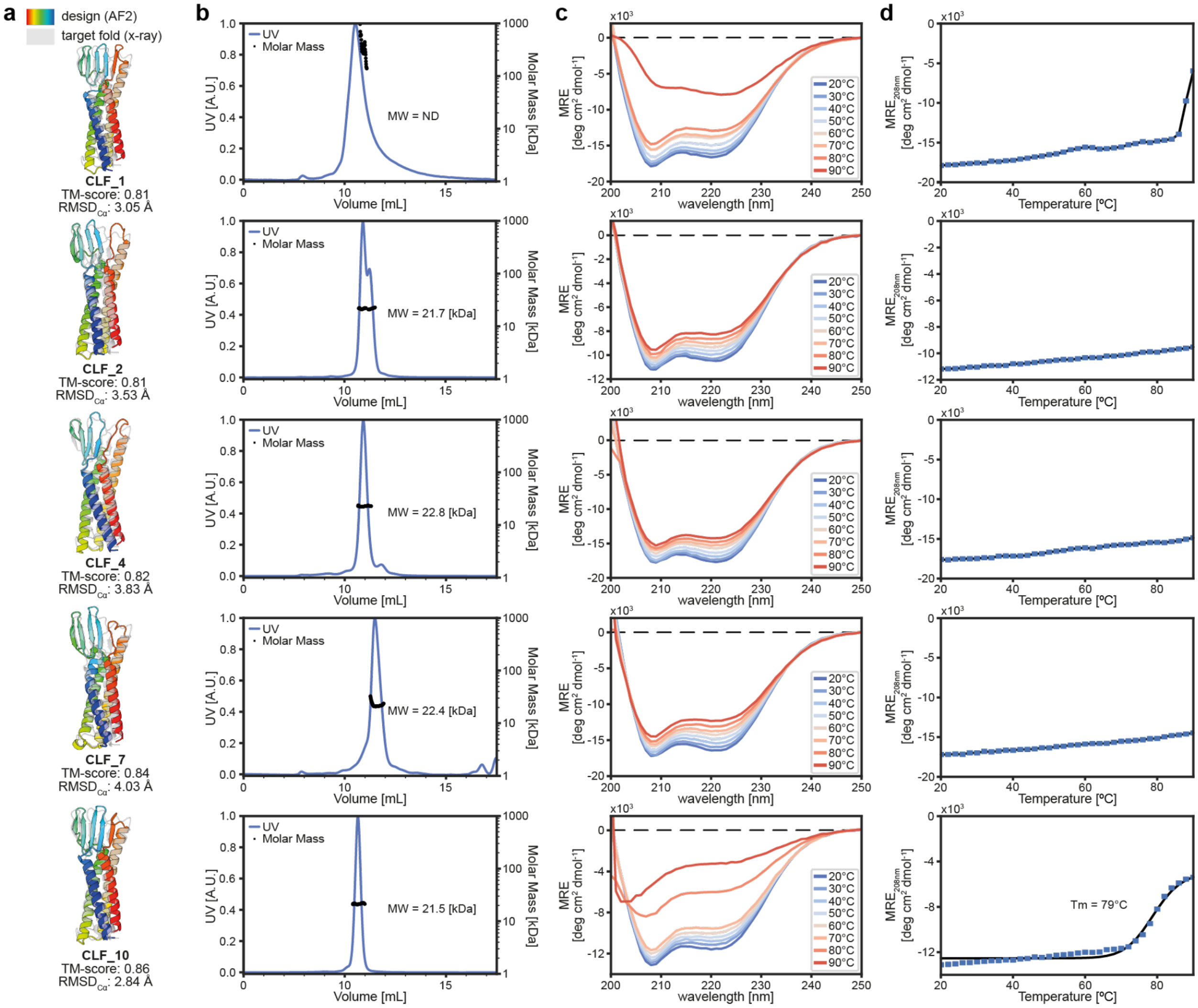

Supplementary Figure 7 | Biophysical characterization of designed Claudin-like folds (CLF). a, Cartoon depiction of design (colored) overlaid on the target fold (gray). **b,** SEC-MALS analysis of corresponding design in panel a. The expected Mw for the monomeric design ranges from 21.5 to 22.5 kDa. **c,** CD spectroscopy measurements at different temperatures. **d,** Thermostability curve based on CD measurement.

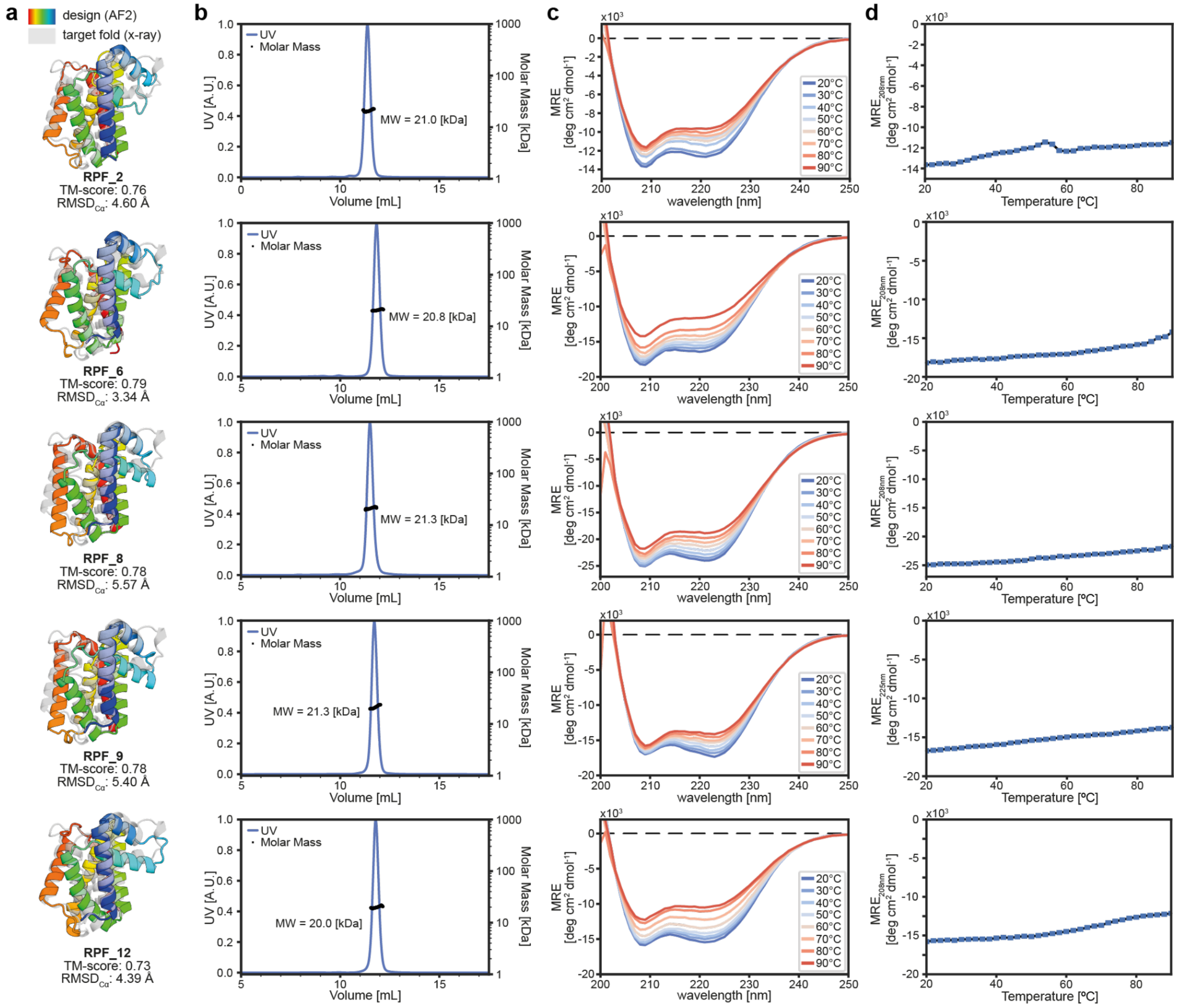

Supplementary Figure 8 | Biophysical characterization of designed Rhomboid protease folds (RPF). **a,** Cartoon depiction of design (colored) overlaid on the target fold (gray). **b,** SEC-MALS analysis of corresponding design in panel a. The expected Mw for the monomeric design ranges from 20.7 to 21.8 kDa. **c,** CD spectroscopy measurements at different temperatures. **d,** Thermostability curve based on CD measurement.

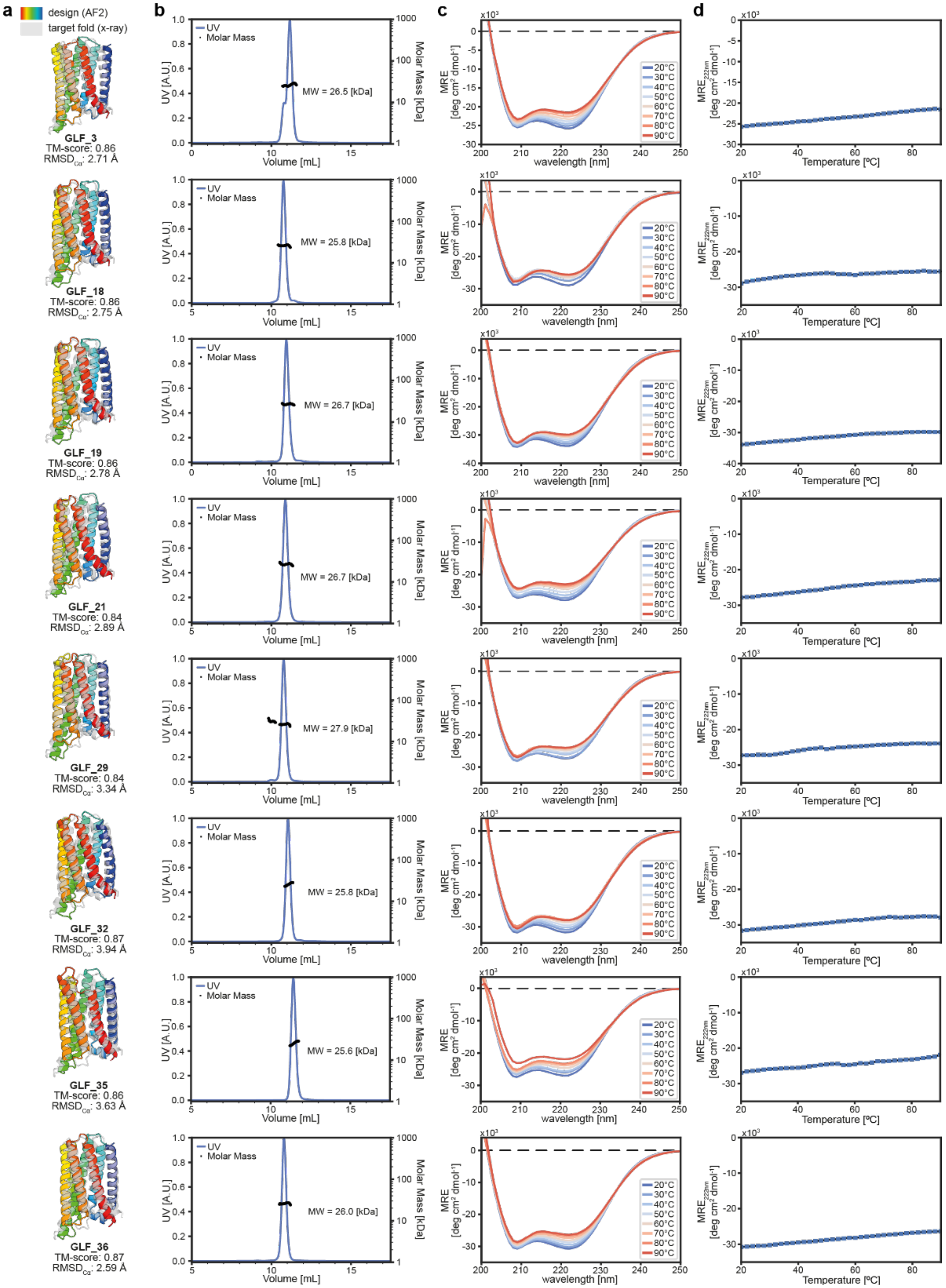
a
design (AF2)
target fold (x-ray)
GLF_3
TM-score: 0.86
RMSD$_{C\alpha}$: 2.71 Å
GLF_18
TM-score: 0.86
RMSD$_{C\alpha}$: 2.75 Å
GLF_19
TM-score: 0.86
RMSD$_{C\alpha}$: 2.78 Å
GLF_21
TM-score: 0.84
RMSD$_{C\alpha}$: 2.89 Å
GLF_29
TM-score: 0.84
RMSD$_{C\alpha}$: 3.34 Å
GLF_32
TM-score: 0.87
RMSD$_{C\alpha}$: 3.94 Å
GLF_35
TM-score: 0.86
RMSD$_{C\alpha}$: 3.63 Å
GLF_36
TM-score: 0.87
RMSD$_{C\alpha}$: 2.59 Å
b
UV
Molar Mass
UV [A.U.]
Molar Mass [kDa]
Volume [mL]
MW = 26.5 [kDa]
MW = 25.8 [kDa]
MW = 26.7 [kDa]
MW = 26.7 [kDa]
MW = 27.9 [kDa]
MW = 25.8 [kDa]
MW = 25.6 [kDa]
MW = 26.0 [kDa]
c
x10³
MRE
[deg cm² dmol⁻¹]
wavelength [nm]
20°C
30°C
40°C
50°C
60°C
70°C
80°C
90°C
d
x10³
MRE$_{222nm}$
[deg cm² dmol⁻¹]
Temperature [ºC]

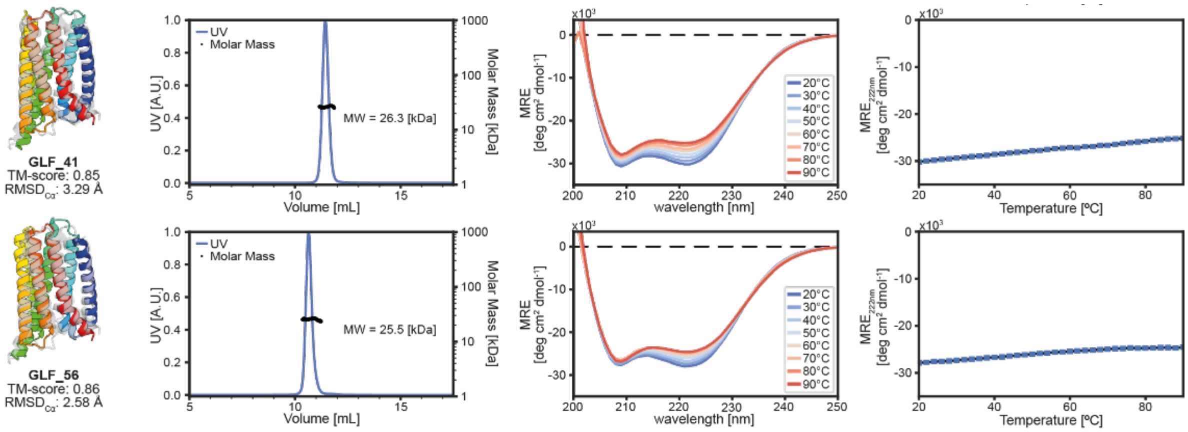

Supplementary Figure 9 | Biophysical characterization of designed GPCR-like folds (GLF). a, Cartoon depiction of design (colored) overlaid on the target fold (gray). **b,** SEC-MALS analysis of corresponding design in panel a. The expected Mw for the monomeric design ranges from 27.2 to 28.1 kDa. **c,** CD spectroscopy measurements at different temperatures. **d,** Thermostability curve based on CD measurements.

a

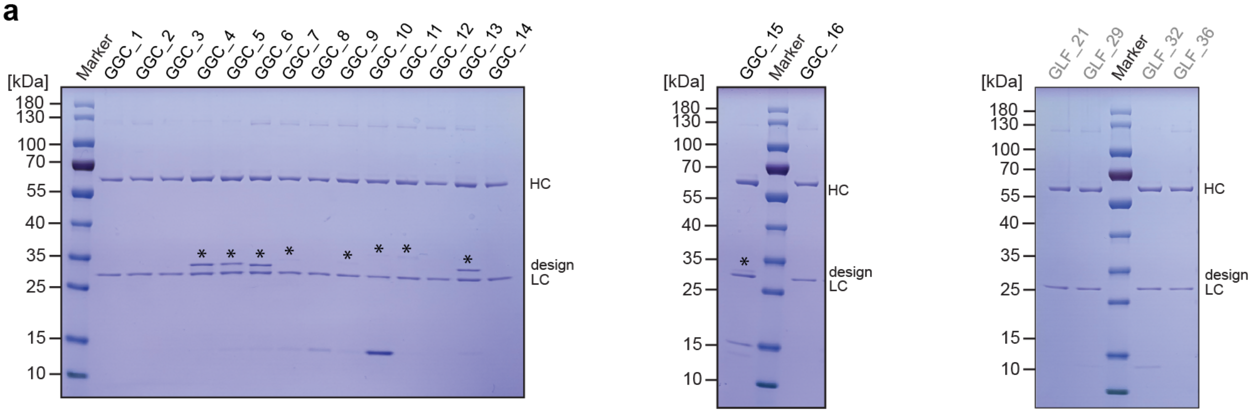

Supplementary Figure 10 | Pulldown screening of antibody binding to GGC designs. a, SDS-PAGE analysis of eluted fractions from a single antibody binding screen to soluble GPCR scaffolds with transplanted ICL3 loops from Ghrelin GPCR receptor. Antibody light-chain (LC) and heavy-chain (HC) are highlighted, design corresponds to soluble GPCR construct. Pulled down constructs are highlighted by asterisks. Soluble scaffolds without transplanted loops are highlighted in gray and serve as negative controls.

Supplementary Table 1 | *In silico* success rates of the design generation.

Fold	Total Designs	Designs Passing Filters	*In silico* success (%)
IGF	150	34	23%
BBF	72	26	36%
TBF	144	84	58%
CLF	750	52	7%
RPF	1769	32	2%
GLF	1063	176	17%
