# Supplementary material for: Computational design of soluble and functional membrane protein analogues
Source: Nature. 2024 Jun 19;631(8020):449–58. doi: 10.1038/s41586-024-07601-y (PMC11236705; doi:10.1038/s41586-024-07601-y)
Supplement: Supplementary file 2 — Reporting Summary [file 41586_2024_7601_MOESM2_ESM.pdf]

nature portfolio

Corresponding author(s): Bruno E. Correia

Last updated by author(s): Apr 16, 2024

Reporting Summary

Nature Portfolio wishes to improve the reproducibility of the work that we publish. This form provides structure for consistency and transparency in reporting. For further information on Nature Portfolio policies, see our Editorial Policies and the Editorial Policy Checklist.

Statistics

For all statistical analyses, confirm that the following items are present in the figure legend, table legend, main text, or Methods section.

n/a	Confirmed	
☐	☒	The exact sample size (*n*) for each experimental group/condition, given as a discrete number and unit of measurement
☐	☒	A statement on whether measurements were taken from distinct samples or whether the same sample was measured repeatedly
☒	☐	The statistical test(s) used AND whether they are one- or two-sided<br>*Only common tests should be described solely by name; describe more complex techniques in the Methods section.*
☒	☐	A description of all covariates tested
☒	☐	A description of any assumptions or corrections, such as tests of normality and adjustment for multiple comparisons
☐	☒	A full description of the statistical parameters including central tendency (e.g. means) or other basic estimates (e.g. regression coefficient) AND variation (e.g. standard deviation) or associated estimates of uncertainty (e.g. confidence intervals)
☒	☐	For null hypothesis testing, the test statistic (e.g. *F*, *t*, *r*) with confidence intervals, effect sizes, degrees of freedom and *P* value noted<br>*Give P values as exact values whenever suitable.*
☒	☐	For Bayesian analysis, information on the choice of priors and Markov chain Monte Carlo settings
☒	☐	For hierarchical and complex designs, identification of the appropriate level for tests and full reporting of outcomes
☒	☐	Estimates of effect sizes (e.g. Cohen's *d*, Pearson's *r*), indicating how they were calculated

Our web collection on statistics for biologists contains articles on many of the points above.

Software and code

Policy information about availability of computer code

Data collection	Chromeleon (ThermoFischer Sci, v7.2.10) and Astra (Wyatt Tech., v8.0.2.5) for collecting SEC-MALS data
Data analysis	Coot (v0.9.5) for structure building; cryoSPARC v4.4.1 was used for cryoEM data processing; UCSF ChimeraX v1.4 and PyMOL (Schrödinger, v2.5.2) for structure visualization; XDS v Jan 10, 2022 (BUILT=20220220) and autoPROC (GlobalPhasing, version 20230222) for crystallographic data processing; Phenix (v1.20.1-4487) for molecular replacement phasing and structure refinement; MolProbity (v4.5.1) for structure validation; Biacore Insight Evaluation Software (Cytiva, v4.0.8.19878) for evaluating surface plasmon resonance measurements; Af2seq code is available at https://github.com/bene837/af2seq. ProteinMPNN, along with soluble trained weights is available at https:// github.com/dauparas/ProteinMPNN.

For manuscripts utilizing custom algorithms or software that are central to the research but not yet described in published literature, software must be made available to editors and reviewers. We strongly encourage code deposition in a community repository (e.g. GitHub). See the Nature Portfolio guidelines for submitting code & software for further information.

Data

Policy information about availability of data

All manuscripts must include a data availability statement. This statement should provide the following information, where applicable:

- Accession codes, unique identifiers, or web links for publicly available datasets
- A description of any restrictions on data availability
- For clinical datasets or third party data, please ensure that the statement adheres to our policy

All data are available in the main text or as supplementary materials. Atomic coordinates and structure factors of the reported X-ray structures have been deposited in the Protein Data Bank under accession numbers 8OYS (TBF_24), 8OYV (CLF_4), 8OYW (RPF_9), 8OYX (GLF_18), and 8OYY (GLF_32). CryoEM model has been deposited in the Protein Data Bank under accession number 9BEI and in the Electron Microscopy Data Bank under entry number 44479. PDB model 7TDM was used for rigid body docking into cryoEM density.

Research involving human participants, their data, or biological material

Policy information about studies with human participants or human data. See also policy information about sex, gender (identity/presentation), and sexual orientation and race, ethnicity and racism.

Reporting on sex and gender	Not applicable to the current study.
Reporting on race, ethnicity, or other socially relevant groupings	Not applicable to the current study.
Population characteristics	Not applicable to the current study.
Recruitment	Not applicable to the current study.
Ethics oversight	Not applicable to the current study.

Note that full information on the approval of the study protocol must also be provided in the manuscript.

Field-specific reporting

Please select the one below that is the best fit for your research. If you are not sure, read the appropriate sections before making your selection.

☒ Life sciences ☐ Behavioural & social sciences ☐ Ecological, evolutionary & environmental sciences

For a reference copy of the document with all sections, see nature.com/documents/nr-reporting-summary-flat.pdf

Life sciences study design

All studies must disclose on these points even when the disclosure is negative.

Sample size	For protein design expression and characterization, 20 designs were tested for IGF, 18 for BBF, 25 for TBF, 57 for GLF, 13 for CLF, 15 for RPF, 16 for GGC, 7 for CLN1, 6 for CLN4, 15 for iGLF, and 15 for aGLF. Designs were chosen according to top scoring in silico prediction metrics.
Data exclusions	Particles were excluded during 2D and 3D classification during cryoEM reconstruction. Removal of suboptimal particles is standard practice in single-particle cryoEM and is necessary to obtain homogeneous reconstructions.
Replication	Solubility expression experiments were performed a single time due to the robustness of expression conditions. SPR binding experiments were measured a single time with multiple concentrations and a negative control in parallel on a separate channel, to rule out unspecific binding events.
Randomization	Randomization is not applicable to this study as no live animals or human subjects were involved.
Blinding	Analyses in this manuscript were not blinded, as no live animals or human subjects were involved. Blinding is not standard practice for the presented in vitro experiments. In silico analyses were automated, no user intervention could introduce bias.

Reporting for specific materials, systems and methods

We require information from authors about some types of materials, experimental systems and methods used in many studies. Here, indicate whether each material, system or method listed is relevant to your study. If you are not sure if a list item applies to your research, read the appropriate section before selecting a response.

Materials & experimental systems

n/a	Involved in the study
☒	☐ Antibodies
☒	☐ Eukaryotic cell lines
☒	☐ Palaeontology and archaeology
☒	☐ Animals and other organisms
☒	☐ Clinical data
☒	☐ Dual use research of concern
☒	☐ Plants

Methods

n/a	Involved in the study
☒	☐ ChIP-seq
☒	☐ Flow cytometry
☒	☐ MRI-based neuroimaging
